# Delayed attendance at routine eye examinations is associated with increased probability of general practitioner referral: a record linkage study in Northern Ireland

**DOI:** 10.1111/opo.12685

**Published:** 2020-04-16

**Authors:** David M Wright, Dermot O'Reilly, Augusto Azuara-Blanco, Raymond Curran, Margaret McMullan, Ruth E Hogg

**Affiliations:** 1 Centre for Public Health, Queen's University Belfast, Belfast, UK; 2 Administrative Data Research Centre – Northern Ireland, Belfast, UK; 3 Health Data Research UK, London, UK; 4 Health and Social Care Board, Belfast, UK

**Keywords:** epidemiology, optometry services, public health

## Abstract

**Purpose:**

To investigate relationships between health and socio-economic status with delayed attendance at routine eye examinations and risk of subsequent general practitioner (GP) referral in Northern Ireland.

**Methods:**

We constructed a cohort of 132 046 community dwelling individuals aged ≥60 years, drawing contextual information from the 2011 Northern Ireland Census. Using linked administrative records of routine eye examinations between 2009 and 2014, we calculated 311 999 examination intervals. Multinomial models were used to estimate associations between contextual factors and examination interval (classified into three groups: early recall, on-time, delayed attendance). Associations between examination interval and referral risk were estimated using logistic regression.

**Results:**

Delayed attendance was recorded for 129 857 (41.6%) examination intervals, 53 759 (17.2%) delayed by ≥6 months. Female sex, poor general or mental health were each associated with delay, as were longer distances to optometry services among those aged ≥70 years (longest vs shortest: Relative Risk Ratio = 1.21 [1.14, 1.28]). Low income and residence in social housing were associated with reduced delay risk. There were 3347 (3.5%) and 11 401 (5.3%) GP referrals in the 60–69 and ≥70 years age groups respectively. Delayed attendance was associated with increased referral risk in both groups (Odds Ratios: 60–69 years = 1.30 [1.04, 1.61]; ≥70 years = 1.07 [1.01, 1.13]).

**Conclusions:**

Poor health and longer distances to optometry services were associated with delayed attendance at routine eye examinations but low income was not. Delayed attendance was associated with increased GP referral risk, indicative of missed opportunities to detect potentially serious eye conditions.

**Supplementary Information:**

The online version of this article (doi:10.1111/opo.12685) contains supplementary material, which is available to authorized users.

## Introduction

Regular eye examinations are recommended for older people to ensure prompt correction of refractive error and to facilitate early detection of major causes of visual impairment including cataract, glaucoma, diabetic retinopathy (DR) and age-related macular degeneration (AMD). Early detection is crucial as severity at presentation is associated with substantially worse visual outcomes and treatment prognoses for glaucoma, AMD^1,2^ and DR.^3^

In the UK, detection of most chronic eye conditions is undertaken by community optometrists at routine free (government funded) eye examinations.^4–6^ On detection of an ocular condition that potentially requires treatment, patients are referred to secondary ophthalmic care, often via their GP (during the period covered by this study, the majority of referrals were via GPs but use of direct referral pathways has increased more recently). Only 7–10% of referrals to GPs are in relation to systemic conditions detected at eye examinations.^7,8^ The majority of referrals are by optometrists^4–6,9^ and so they can be considered the gatekeepers to eye care in the UK. Optimising use of these services could have a profound impact on population eye health and reduce care costs stemming from delayed diagnosis of chronic conditions.

The work of community optometrists is governed by clinical guidelines indicating recommended minimum intervals for eye examinations, based on age and presence of established risk factors for specific conditions (diabetic retinopathy and glaucoma). In England, Wales and Northern Ireland (NI), those aged ≥60 years and with no other risk factors are eligible for examinations biannually and those aged ≥70 are eligible for annual examinations,^10^ as are those aged >40 with a family history of glaucoma. However, uptake frequently falls short of clinical guidelines.^11^ This may be partially due to limited public understanding of eye diseases and the importance of regular examinations, with attendance often prompted by onset or worsening of symptoms.^12,13^ Qualitative studies indicate that costs (either perceived or actual) may be a barrier to uptake,^12,14^ but evidence for an association between uptake and socio-economic status (SES) is equivocal.^15^ Uptake can vary with area of residence^16^ and a recent UK study found that long distances to the nearest optometric practice and lack of car transport were associated with reduced uptake of eye examinations among older people.^17^ Other variables associated with uptake included sex, household structure and certain chronic health conditions (e.g. cognitive impairment). Little is known about the extent to which uptake (and social gradients in uptake) influences clinical eye health outcomes at the population scale and in particular, whether delayed attendance at routine eye examinations has a measurable impact on eye health.

We used records of all publicly funded eye examinations attended by those aged ≥60 years within NI to (1) describe the social patterning of intervals between routine eye examinations and (2) investigate whether delayed attendance is associated with increased risk of referral to a GP (a proxy for referral to secondary ophthalmic care).

## Methods

### Data sources

Information on attendance at routine eye examinations was drawn from the Family Practitioner Services Ophthalmic Database (managed by the Business Services Organisation, NI Department of Health), an administrative database used to manage payment to service providers. Records of eye examinations of those aged ≥60 years conducted during a 5-year period (October 2009 to September 2014 inclusive) were extracted. This period coincided with strategic efforts to improve commissioning and provision of eye care in NI.^18^ Information on individual and household characteristics was drawn from the 2011 Census. Both the 2011 Census and the Ophthalmic Database are of sufficient quality for the production of government statistics (including National Statistics for the Census).^19,20^

### Cohort construction

**Figure 1:**
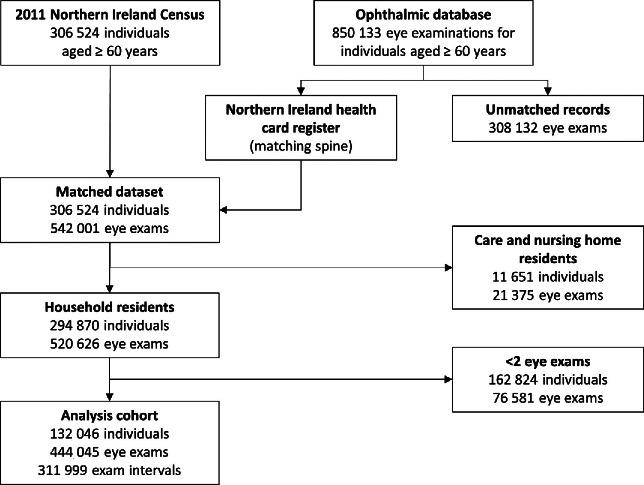
Construction of the linked dataset comprising records from the 2011 Northern Ireland Census and the ophthalmic database.

Cohort construction is summarised in *Figure* *1*. Ophthalmic and Census datasets were linked using the NI health card registration system, a register containing address histories for those accessing primary healthcare services. Linkages were made using a series of deterministic match keys validated for these data (e.g. name, address and date of birth). Eye examinations were matched to an individual at any of the addresses at which they had lived during the study period, generating a longitudinal sequence of examination records for each individual. Each individual sequence was then matched to a Census record. In this way the sequence of events both before and after the Census was reconstructed, even if individuals had changed address. Linkages were made within the Administrative Data Research Centre-NI. To protect individual privacy, records were de-identified before the researchers accessed the linked dataset. Ethical approval was received from the UK National Research Ethics Service (reference: 16/EM/0103). The cohort consisted of all community-dwelling respondents to the 2011 Census aged ≥60 years at the beginning of the study period that had attended at least two free eye examinations during the period (132 046 individuals from 111 380 households). 93.8% of the cohort survived the study period. A longitudinal sequence of eye examinations was constructed for each individual and from these, the analysis dataset of 311 999 examination intervals was calculated (*Figure* *1*).

### Outcome variables

We classified examination intervals into three categories based on recommended intervals for each age group. The first category ‘on-time’, consisted of intervals conforming to recommendations (24 months for those aged 60–69, 12 months for those aged ≥70). Longer intervals (>24 and >12 months for the younger and older groups respectively) were classified as ‘delayed attendance’. The third category ‘early recall’, consisted of intervals shorter than recommended. Early recall may occur when an individual returns to the optometrist with visual symptoms or at the request of the optometrist to monitor an ocular condition that does not warrant immediate GP referral. Therefore, we expected that this category would have higher referral risk than the other two. Year and month of each examination were available (full dates were unavailable due to the potential for disclosure) and so classifications were based on calculations in calendar months. For example, the next examination for a 75-year old individual examined in February 2011 would have been due in February 2012. If the second examination was recorded in March 2012 then it would have been classed as delayed (or early if recorded in January 2012). Therefore, our definition of early recall corresponds with that used for service provision up to the accuracy of the available data. Payment regulations for examinations specify intervals in days, with clinical justification required for examinations conducted more than 2 weeks earlier than the recommended minimum interval.

### Other variables

Age, sex, ethnicity, religion, highest educational attainment and self-reported health status were drawn from 2011 NI Census returns. Extent of caregiving responsibilities in terms of hours of unpaid care provided per week was also reported. At the household level, housing tenure, car access and whether accommodation was adapted for visual impairment (yes/no) were selected along with a classification of household structure (e.g. living alone/living with a partner). We also selected an area-based measure of SES, the proportion of the population living in households in receipt of income-related benefits or tax credits.^21^ Drive time to the nearest optometry practice and density of optometry practices in the local area were used as measures of eye care accessibility and availability.^17^ Individual eligibility for free examinations on low income (evidenced by receipt of certain benefits) or health grounds (glaucoma; glaucoma risk factors or relative with glaucoma; diabetes; complex health needs; registered blind) in addition to age eligibility were extracted from examination records. The Census records used in this study had undergone extensive quality checks as part of the standard Census procedure, which included imputation of missing values for unanswered questions to produce a complete record for each individual. The two eligibility variables were derived from a broader set of checkboxes specifying grounds for free eye examination. Age eligibility alone was sufficient so in some cases, relevant boxes may have been left unchecked, leading to underestimation of eligibility for non-age reasons that we have not attempted to adjust for.

### Statistical analysis

Our analysis had two stages, first estimating associations between individual and household characteristics and eye examination interval, and second, estimating associations between examination interval and risk of GP referral. We modelled the associations between predictor variables and examination intervals using multinomial models to simultaneously estimate relative risks of delayed attendance or early recall, using the on-time group as the reference category. Multivariable models containing all predictor variables were fitted separately for each age group. Associations between examination interval and GP referral risk were estimated using logistic regression. We fitted additional models replacing the three-category classification of examination intervals with the actual interval lengths in months to determine whether there were systematic changes in referral risk as attendance delay increased (e.g. a linear increase in referral risk with each additional month of delay). All models were fitted using R software (www.R-project.org).^22^

## Results

### Examination intervals

Delayed attendance was recorded for a total of 129 857 (41.6%) examination intervals, 53 759 (17.2%) delayed by ≥6 months. In both age groups the number of delayed eye examinations exceeded the number of on-time examinations (*Table* *1*). In the 60–69 age group, short examination intervals (early recall) were most common but in the ≥70 years group they had similar frequency to on-time examinations. Distribution of exam intervals was multimodal with the highest peak in attendance at 12 months for both age groups and smaller peaks at 6 and 24 months (Figure S1), reflecting standard recall intervals used by optometrists based on assessment of clinical need (guidelines allow flexible recall intervals). Six-, 12- and 24-month intervals constituted 31% of the total.

**Table 1:** Association between routine eye examination interval and risk of General Practitioner referral by age group

Examination interval	Aged 60–69			Aged ≥ 70		
	Total	% referred	OR (95% CI) referred^a^	Total	% referred	OR (95% CI) referred^a^
On-time	7311	1.5	1.00	54 770	3.8	1.00
Delayed attendance	17 822	1.9	1.30 (1.04, 1.61)	112 035	4.0	1.07 (1.01, 1.13)
Early recall	69 853	4.1	2.86 (2.36, 3.46)	50 208	9.6	2.72 (2.58, 2.87)

Totals are number of examination intervals not number of individuals.

CI, Confidence Interval; OR, Odds Ratio.

^a^ Highlighting indicates cells with 95% Confidence Intervals not overlapping the reference value (blue = below reference; orange = above reference).

*Table* *2* shows the social patterning of delayed attendance. Only those variables most strongly associated with delayed attendance (relative risk ratio differentials >10% between at least two variable levels) are displayed. Estimates for other variables are given in *Table* S1. Corresponding estimates for early recall are given in *Table* *3* and *Table* S2.

**Table 2:** Individual and household characteristics and associations between characteristics and delayed attendance at routine eye examinations, 2009–2014, among those aged 60 years and over in Northern Ireland, UK

Variable	Level	Aged 60–69			Aged ≥ 70		
		Total (*N* = 94 986) (%)	Delayed (%)	Adjusted RRR (95% CI)^a^	Total (*N* = 217 013) (%)	Delayed (%)	Adjusted RRR (95% CI)^a^
Sex	Females	56.8	18.4	1.00	59.3	52.2	1.00
	Males	43.2	19.2	0.87 (0.82, 0.92)	40.7	50.8	0.82 (0.80, 0.84)
Religion	Protestant^b^	64.4	18.4	1.00	69.0	51.0	1.00
	Catholic	33.2	19.5	1.12 (1.05, 1.19)	29.4	53.0	1.14 (1.12, 1.17)
	No religion	1.5	19.7	1.13 (0.90, 1.41)	0.8	53.5	1.18 (1.06, 1.33)
	Other religions	0.9	16.3	1.30 (0.92, 1.84)	0.7	49.8	1.07 (0.95, 1.21)
Eligibility-income grounds	No	73.9	19.9	1.00	71.3	52.8	1.00
	Yes	26.1	15.6	0.79 (0.73, 0.85)	28.7	48.7	0.86 (0.84, 0.89)
Eligibility-health grounds	No	98.8	18.9	1.00	99.1	51.7	1.00
	Yes	1.2	8.3	1.34 (0.87, 2.06)	0.9	44.2	0.81 (0.73, 0.91)
General health	Very good	18.8	21.8	1.00	12.9	53.6	1.00
	Good	36.8	20.1	1.04 (0.96, 1.12)	35.7	51.7	1.01 (0.98, 1.05)
	Fair	30.8	17.1	1.12 (1.02, 1.23)	40.9	51.0	1.09 (1.05, 1.13)
	Bad	11.1	14.9	1.22 (1.06, 1.40)	8.9	51.3	1.20 (1.14, 1.26)
	Very bad	2.5	14.5	1.17 (0.94, 1.47)	1.7	51.2	1.25 (1.14, 1.37)
Health condition	Memory loss	2.7	14.7	0.97 (0.80, 1.18)	3.7	48.7	0.98 (0.92, 1.04)
	Learning diff.	0.7	14.8	0.68 (0.49, 0.95)	0.4	52.2	1.12 (0.94, 1.34)
	Communication diff.	1.0	14.5	1.22 (0.89, 1.69)	1.2	48.5	1.01 (0.91, 1.12)
	Mental condition	8.0	17.3	1.17 (1.05, 1.31)	3.4	52.3	1.08 (1.02, 1.15)
	Blind/part. sight	3.5	10.4	1.11 (0.91, 1.37)	6.8	45.0	0.95 (0.91, 1.00)
	Mobility diff.	28.1	15.9	0.96 (0.88, 1.05)	37.2	50.5	1.00 (0.98, 1.03)
	Chronic illness	21.4	12.8	1.13 (1.04, 1.23)	23.5	49.9	0.91 (0.89, 0.94)
	Breathing diff.	16.5	16.0	0.93 (0.86, 1.01)	18.9	51.2	1.06 (1.03, 1.09)
	Deaf/hearing impairment	12.0	16.9	1.01 (0.92, 1.10)	23.8	49.5	0.97 (0.95, 1.00)
	Chronic pain	27.4	16.7	1.02 (0.94, 1.10)	28.5	51.1	1.01 (0.98, 1.04)
	Other chronic condition	10.1	17.1	0.99 (0.90, 1.09)	9.4	51.9	1.03 (0.99, 1.06)
	No condition	89.9	18.9	1.00	90.6	51.6	1.00
Household structure	Alone	20.2	16.0	1.00	34.4	49.1	1.00
	Partner only	50.0	19.1	1.07 (0.99, 1.16)	44.8	52.7	1.11 (1.09, 1.14)
	Partner, children	18.0	21.3	1.16 (1.06, 1.28)	7.9	55.7	1.20 (1.15, 1.25)
	Partner, others	1.3	18.9	1.24 (0.96, 1.61)	0.8	56.4	1.26 (1.12, 1.42)
	Children only	4.4	17.7	1.00 (0.86, 1.15)	6.1	51.9	1.07 (1.02, 1.12)
	Siblings only	1.5	15.7	1.00 (0.78, 1.28)	1.9	49.5	0.92 (0.86, 1.00)
	Complex/other	4.6	19.3	1.09 (0.94, 1.25)	4.1	53.3	1.13 (1.07, 1.20)
Tenure	Owner occupied	80.1	19.6	1.00	77.3	52.5	1.00
	Private rented	4.9	17.9	1.06 (0.93, 1.21)	4.6	50.8	1.03 (0.98, 1.08)
	Social rented	12.8	14.7	0.78 (0.71, 0.86)	13.1	48.4	0.88 (0.85, 0.91)
	Rent free	2.2	15.3	0.85 (0.70, 1.03)	5.0	47.4	0.87 (0.83, 0.91)
Household cars	One or more	86.1	19.3	1.00	75.4	52.2	1.00
	None	13.9	15.4	0.83 (0.76, 0.91)	24.6	49.8	1.00 (0.97, 1.03)
Practice density (per 1000)	0	81.2	18.9	1.00	79.7	52.0	1.00
	(0.01,1]	14.5	18.1	1.03 (0.95, 1.12)	15.3	51.4	1.02 (0.99, 1.05)
	(1,4.57]	4.3	18.2	0.97 (0.85, 1.12)	4.9	47.1	0.90 (0.86, 0.94)
Drive time (min)	[0,2)	45.6	18.1	1.00	48.2	50.7	1.00
	[2,4)	25.5	19.0	1.04 (0.97, 1.12)	25.3	51.4	1.03 (1.01, 1.06)
	[4,6)	12.2	19.5	1.08 (0.98, 1.18)	11.2	53.3	1.11 (1.07, 1.15)
	[6,8)	7.6	19.2	1.11 (0.99, 1.24)	7.1	53.3	1.10 (1.05, 1.14)
	[8,10)	5.1	20.5	1.23 (1.07, 1.41)	4.4	54.0	1.13 (1.07, 1.19)
	[10,20)	4.1	19.8	0.99 (0.86, 1.14)	3.8	54.9	1.21 (1.14, 1.28)

Multivariate estimates are adjusted for all variables in the table and highest qualification; carer status; household adaptations for visual difficulties; area income deprivation.

CI, Confidence Interval; RRR, Relative Risk Ratio.

^**a**^Highlighting indicates cells with 95% Confidence Intervals not overlapping the reference value (blue = below reference; orange = above reference).

^b^ And Other Christian (including Christian Related).

**Table 3:** Individual and household characteristics and associations between characteristics and early attendance at routine eye examinations, 2009–2014, among those aged 60 years and over in Northern Ireland, UK

Variable	Level	Aged 60–69			Aged ≥ 70		
		Total (*N* = 94 986) (%)	Early (%)	Adjusted RRR (95% CI)^a^	Total (*N* = 217 013) (%)	Early (%)	Adjusted RRR (95% CI)^a^
Sex	Females	56.8	74.4	1.00	59.3	23.9	1.00
	Males	43.2	72.5	0.78 (0.74, 0.82)	40.7	22.0	0.81 (0.78, 0.83)
Religion	Protestant^b^	64.4	74.0	1.00	69.0	23.2	1.00
	Catholic	33.2	72.6	0.98 (0.92, 1.03)	29.4	22.9	1.08 (1.05, 1.11)
	No religion	1.5	72.7	0.99 (0.81, 1.21)	0.8	23.0	1.10 (0.96, 1.26)
	Other religions	0.9	78.5	1.54 (1.12, 2.10)	0.7	26.5	1.23 (1.07, 1.41)
Eligibility-income grounds	No	73.9	72.6	1.00	71.3	22.3	1.00
	Yes	26.1	76.2	0.96 (0.90, 1.02)	28.7	25.1	1.07 (1.03, 1.10)
Eligibility-health grounds	No	98.8	73.4	1.00	99.1	23.1	1.00
	Yes	1.2	89.2	3.48 (2.37, 5.11)	0.9	29.1	1.19 (1.06, 1.35)
General health	Very good	18.8	69.3	1.00	12.9	19.8	1.00
	Good	36.8	71.9	1.12 (1.05, 1.19)	35.7	22.4	1.16 (1.11, 1.21)
	Fair	30.8	75.8	1.29 (1.18, 1.40)	40.9	24.3	1.29 (1.23, 1.35)
	Bad	11.1	78.8	1.44 (1.27, 1.63)	8.9	25.3	1.41 (1.33, 1.51)
	Very bad	2.5	79.0	1.33 (1.09, 1.62)	1.7	26.4	1.51 (1.36, 1.68)
Health condition	Memory loss	2.7	78.7	1.03 (0.87, 1.22)	3.7	27.2	1.09 (1.02, 1.16)
	Learning diff.	0.7	74.9	0.60 (0.46, 0.80)	0.4	23.6	0.92 (0.75, 1.13)
	Communication diff.	1.0	79.4	1.29 (0.98, 1.71)	1.2	27.6	1.08 (0.96, 1.21)
	Mental condition	8.0	75.9	1.02 (0.92, 1.13)	3.4	24.2	1.00 (0.94, 1.07)
	Blind/part. sight	3.5	85.5	2.09 (1.75, 2.50)	6.8	31.7	1.42 (1.36, 1.49)
	Mobility diff.	28.1	77.2	1.04 (0.97, 1.12)	37.2	25.2	1.10 (1.06, 1.13)
	Chronic illness	21.4	82.3	1.93 (1.79, 2.08)	23.5	24.1	0.96 (0.93, 0.99)
	Breathing diff.	16.5	76.8	0.95 (0.88, 1.02)	18.9	24.6	1.05 (1.01, 1.08)
	Deaf/hearing impairment	12.0	76.2	1.08 (1.00, 1.17)	23.8	25.4	1.08 (1.05, 1.11)
	Chronic pain	27.4	76.4	0.99 (0.93, 1.06)	28.5	24.6	0.98 (0.95, 1.01)
	Other chronic condition	10.1	75.8	1.10 (1.01, 1.19)	9.4	24.1	1.03 (0.99, 1.08)
	No condition	89.9	73.3	1.00	90.6	23.0	1.00
Household structure	Alone	20.2	76.1	1.00	34.4	24.8	1.00
	Partner only	50.0	73.3	0.94 (0.88, 1.01)	44.8	22.4	0.95 (0.92, 0.98)
	Partner, children	18.0	70.9	0.90 (0.83, 0.98)	7.9	20.2	0.89 (0.84, 0.94)
	Partner, others	1.3	74.5	1.11 (0.88, 1.40)	0.8	20.1	0.90 (0.78, 1.04)
	Children only	4.4	74.1	0.88 (0.78, 1.00)	6.1	23.6	0.94 (0.89, 0.99)
	Siblings only	1.5	77.1	1.09 (0.88, 1.35)	1.9	23.3	0.89 (0.81, 0.97)
	Complex/other	4.6	72.8	0.91 (0.80, 1.03)	4.1	23.0	0.94 (0.88, 1.01)
Tenure	Owner occupied	80.1	73.0	1.00	77.3	22.9	1.00
	Private rented	4.9	74.8	1.02 (0.91, 1.15)	4.6	24.8	1.06 (1.00, 1.13)
	Social rented	12.8	76.2	0.85 (0.79, 0.93)	13.1	23.3	0.88 (0.85, 0.92)
	Rent free	2.2	76.5	0.95 (0.80, 1.11)	5.0	25.5	0.97 (0.92, 1.03)
Household cars	One or more	86.1	73.2	1.00	75.4	22.9	1.00
	None	13.9	75.5	0.85 (0.79, 0.93)	24.6	23.8	0.90 (0.87, 0.93)
Practice density (per 1000)	0	81.2	73.4	1.00	79.7	23.0	1.00
	(0.01,1]	14.5	74.2	1.06 (0.98, 1.14)	15.3	23.0	1.01 (0.97, 1.04)
	(1,4.57]	4.3	73.3	0.95 (0.84, 1.07)	4.9	25.6	1.06 (1.01, 1.13)
Drive time (min)	[0,2)	45.6	73.9	1.00	48.2	23.3	1.00
	[2,4)	25.5	73.4	1.01 (0.95, 1.08)	25.3	23.6	1.04 (1.01, 1.07)
	[4,6)	12.2	73.1	1.02 (0.94, 1.11)	11.2	22.9	1.04 (1.00, 1.09)
	[6,8)	7.6	73.6	1.08 (0.97, 1.19)	7.1	22.2	0.98 (0.93, 1.03)
	[8,10)	5.1	72.7	1.11 (0.98, 1.25)	4.4	22.1	0.99 (0.93, 1.06)
	[10,20)	4.1	72.0	0.94 (0.83, 1.06)	3.8	22.5	1.07 (1.00, 1.15)

Multivariate estimates are adjusted for all variables in the table and highest qualification; carer status; household adaptations for visual difficulties; area income deprivation.

CI, Confidence Interval; RRR, Relative Risk Ratio.

^a^ Highlighting indicates cells with 95% Confidence Intervals not overlapping the reference value (blue = below reference; orange = above reference).

^b^ And Other Christian (including Christian Related).

Eligibility for free eye examinations on low income grounds was associated with decreased risk of delayed attendance, adjusting for other factors. Delayed attendance was less likely among males than females (13% and 18% in 60–69 and ≥70 years groups respectively). Eligibility on health grounds was rarely reported (<2% in each age group) and was associated with delayed attendance only among those aged ≥70 years. There was a gradient of increasing delay risk with worsening general health in both age groups (25% greater risk: very bad health vs very good health in the older group). Poor mental health was associated with delayed attendance in both age groups but associations with other chronic conditions were not consistent across groups.

Residence in social rented accommodation was associated with decreased delay risk in both age groups. Household structure was important for the older group; living with others as opposed to living alone was associated with increased risk of delayed attendance, with the exception of living with siblings only. These associations were less clear in the younger group. There was an urban/rural gradient in delayed attendance in the older group only. Living in areas with the highest density of optometry practices (i.e. urban area) was associated with decreased delay risk. Risk of delayed attendance increased with drive time to the nearest practice in the older group only. There were no consistent gradients in delayed attendance with area level income deprivation in either age group.

### GP referrals

There were totals of 3347 (3.5%) and 11 401 (5.3%) GP referrals in the 60–69 and ≥70 years age groups respectively. Delayed attendance was associated with increased referral risk in both groups (*Table* *1*) with a proportionately larger increase in relative risk in the 60–69 group than in the ≥70 years group (30% and 7% respectively). As expected, early attendance was associated with more than two-fold increase in referral risk for both groups.

**Figure 2:**
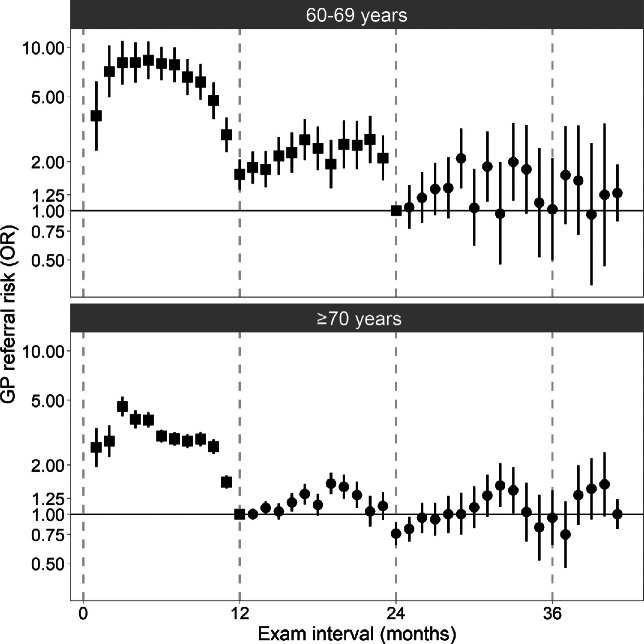
Risk of General Practitioner referral following routine eye examinations among those aged ≥60 years, Northern Ireland, UK. Odds ratios and 95% confidence intervals given. Squares indicate early or on-time attendance, circles indicate delayed attendance.

The month level analysis revealed complex relationships between examination interval and GP referral risk with increasing time beyond recommended intervals. Among those aged 60–69 there was a gradual increase in risk with increasing delay (*Figure* *2*). By 5 months (29-month interval) referral risk had increased twofold (OR = 2.09; CI: 1.37, 3.18) and there was strong evidence for a risk differential (i.e. 95% CI not overlapping baseline). There was no clear pattern in referral risk with further delays; point estimates for the majority of subsequent months were above baseline, some showing a twofold increase in risk. Among those aged ≥70 years there was a similar pattern of increasing risk with increasing delay, peaking at 7 months beyond the 12 month recommended examination interval (OR = 1.47; CI: 1.24, 1.7; *Figure* *2*). Risk decreased for the next 5 months with a minimum at 24 months substantially below baseline (OR = 0.76; 95% CI: 0.65, 0.89). The pattern of substantial increase and decrease was repeated in the 24–36 month period.

## Discussion

### Factors associated with delayed attendance

Poor general health and chronic mental ill-health were both associated with delayed attendance at routine eye examinations, indicating that systemic health conditions may be a barrier to attendance, perhaps through increased difficulties arranging and travelling to appointments.^23^ Poor health may also have contributed towards the increased delay risk among those aged ≥70 years and living with children, an indicator of frailty in this age group. Poor health was not a major theme in qualitative studies of barriers to eye test uptake,^12,14^ perhaps because those with poor health may have been less likely to attend focus-group discussions than healthy individuals.

Women were more likely to delay attendance but were also more likely to be recalled early than men, indicating greater risk of eye conditions requiring regular monitoring. This accords with global estimates indicating that prevalence of eye conditions is greater among women than men.^24,25^ From a population health perspective, the risks associated with delayed attendance among women may be no greater than those associated with lower overall uptake of eye examinations among men.^17^ Women may have higher risk of eye conditions and be more likely to delay attendance but men are less likely to attend routine eye examinations at all, potentially presenting with more severe disease when a condition is eventually detected. Further research would be required to determine how these factors interact to influence the overall burden of disease in terms of the number and severity of cases.

Those with low income (either eligible for eye examinations on income grounds or resident in social housing) were less likely to be delayed than the more affluent. Measures of socio-economic status were not associated with overall uptake^17^ so we find little evidence that low SES in itself is a barrier to eye care in this population among those aged over 60 years. This may be because those in receipt of income-related benefits are eligible for vouchers to substantially offset the cost of spectacles or contact lenses, potentially reducing the perceived cost of attending an eye examination. These findings contrast with those from other parts of the UK. A Scottish study indicated that low income was associated with substantially lower eye examination uptake, a gradient exacerbated by extension of eligibility for free eye examinations to all age groups.^26^ Similarly, low uptake of free eye examinations in the most deprived areas was reported in an area-based study in Leeds, England.^16^ We have no simple explanation for this discrepancy.

Greater drive time to the nearest optometrist, a proxy for rural residence in this dataset, was associated with increased delay risk among those aged ≥70 years, even after adjustment for lower SES in rural areas. Taken together with our previous finding that overall uptake decreased with increasing drive time,^17^ this indicates that geographical accessibility of services is an important determinant of eye care access in this group.

The identified factors were associated with modest variation in risk of delayed attendance in absolute terms. For example, there was a 7.3% differential in risk between those with very bad health and those with very good health among those aged 60–69. The differential between women and men was 1.2% in the 60–69 group and 1.4% among those aged ≥70 years. However, given the size of the respective groups, even small differences in absolute rates could translate into large differences in the total number of GP referrals.

### Delayed attendance and GP referral

Our study is the first to assess the influence of a delay in routine eye examinations on health outcomes at the population level using linked administrative data. We found that risk of GP referral among those aged ≥60 years increased with delays in attendance at routine eye examinations and that by 6 months beyond recommended examination intervals, risk had increased substantially (one and a half to twofold) in an approximately linear fashion. There was no clear pattern in referral risk with further delay. Our results also indicate that the current system of recall, in which intervals are flexible according to optometrists' clinical judgement is broadly appropriate. For example, the large number of eye examinations at 24 months interval among those aged ≥70 years likely represents individuals that were recommended a longer interval because they were in good health and showed no signs of ocular conditions at the initial examination. Accordingly, this group had substantially reduced referral risk than those on the standard 12-month interval. Similarly, there was a large number of 12-month intervals (early attendance) among those in the 60–69 group that was associated with increased referral risk compared to those on the standard 24-month interval. Whilst we cannot directly determine whether interval lengths were patient-initiated or optometrist suggested, peaks in the interval distribution (Figure S1) at 6, 12 and 24 months are likely to represent optometrist suggested intervals and constitute a substantial proportion of the total.

### Strengths and limitations

The main strength of this study was dataset size (an order of magnitude larger than previous studies), enabling us to examine the complex multimodal relationships between examination interval and GP referral in each age group. Each month level estimate of referral risk was supported by considerable data. The minimum number of intervals for a given month was 216 (40 months, 60–69 age group) and for those aged ≥70 years each estimate between 12 and 24 months corresponded to more than 2300 intervals, giving ample degrees of freedom for estimation. Therefore, it is likely that these estimates provide a good representation of the patterns of variation in outcomes.

Use of administrative data gave information on service use across all societal strata and linkage to the Census provided rich contextual information with low risk of response bias (the 2011 Census had a response rate >95% for this age group). An advantage of using the ophthalmic database was that data collection was uniform across the study population with records satisfying defined quality standards. For example, date of examination was a mandatory field for payment approval and so it was in the interests of practices to report it accurately. Therefore, measurement of examination intervals is likely to have been of similar accuracy across the dataset. Also, boundaries for the early/on-time/delayed classification were based on clearly defined clinical guidelines; a valid clinical reason for an early recall must be given, regardless of whether the examination is initiated by a patient presenting with symptoms or the early recall has been suggested by the optometrist at a previous examination. Consequently, the higher ratio of early recall to on-time examinations in the 60–69 age group compared with the ≥70 age group is unlikely to have resulted from measurement error or misclassification at the practice level. One potential explanation for the higher ratio in the 60–69 age group is that scheduling an examination 2 years in advance (especially among those still in employment) is more difficult than scheduling 1 year ahead. Furthermore, in the 60–69 age group a greater proportion of people were in good health and so less likely to be entrained into the regular schedules of health checks that become more common as symptoms appear with age.

However, scheduling difficulties alone are unlikely to fully explain the much greater proportion of early recalls in the 60–69 group. This may also indicate that optometrists consider the standard 2-year interval too long in many cases and specify a shorter interval (usually a year) instead. There is some justification for this as the referral risk following early recall was more than double that following on-time examination. Whether the cost of these additional examinations is justifiable in population health terms remains to be determined. Formal analysis using techniques from health economics would provide insight into whether target referral rates should be set and if so, what those targets should be. Such a study might also consider the relative merit of attempting to increase uptake among those with no recorded eye examinations, who did not feature in this analysis.

A limitation was that the recall interval requested by the optometrist was not recorded in the ophthalmic database. A substantial proportion of those aged ≥70 years attending at a 24-month interval and classified as delayed attenders in our analysis were likely to have been adherent to the clinical recommendations of their optometrist. This misclassification likely caused an underestimation of the effect of delay among those in this age group, potentially explaining why the elevated risk associated with delayed attendance was less pronounced than among those aged 60–69 years. The low income indicator based on eligibility recorded in the ophthalmic database probably excludes a proportion in this group, as age was the default criterion recorded for each examination and the system did not require all relevant criteria to be recorded. However, similar patterns of attendance were observed for our other measure of income (housing tenure) so we believe our conclusions are justified. During the study period, referrals to eye casualty, private ophthalmology services and rapid access referrals to macular clinics (for wet AMD) would not have been routed through GPs. We were unable to quantify these referrals as secondary care data is currently unavailable for linkage to the other datasets but we expect the numbers to have been relatively small. A survey of NI optometrists in 2014 reported that only 2.2% of referrals were for wet AMD.^7^ Finally, as this is a cross-sectional study we are limited to discussing associations between factors rather than advancing causal explanations.

### Implications and further work

Long delays in attendance at routine eye examination may have serious implications for individual patients. The most common reasons for referrals from optometrists to GPs in NI are cataracts, anterior eye problems and suspected glaucoma, constituting 50% of the total referrals.^8^ Prognosis for glaucoma patients is improved substantially by prompt access to treatment^27^ and the same is true for those with wet AMD.^28^ At the population level, large numbers of examinations were delayed by more than 6 months (>50 000; 17%). Therefore, even a modest decrease in the proportion of examinations delayed could hasten diagnosis and treatment for a large number of people with suspected eye conditions.

Our findings suggest possible target groups for intervention to encourage on-time attendance at routine eye examinations, namely those with poor general or mental health and those aged ≥70 years living far from the nearest optometry practice. Distance to services was highlighted as an important predictor of any eye examination uptake in this population^17^ and so this should be viewed as a key factor.

To explore directly the implications of delayed eye examination attendance, a possible extension to this work would be to link Census and routine eye examination records to secondary eye care data. This would enable direct measurement of the combined influence of low or delayed eye examination uptake on severity at presentation and prognosis for specific conditions (e.g. AMD, glaucoma) and hence calculation of the burden of eye disease that might be avoided by encouraging timely service uptake. The findings that females were more likely to be delayed and that those with low income were less likely to be delayed were unexpected and would be of research interest, given the contrasting results of SES and eye examination uptake studies in other parts of the UK.

## Conclusion

Using a large linked dataset we have shown that poor general or mental health were associated with delayed attendance at routine eye examinations among those aged ≥60 years, but low income was not. Longer distances to optometry services were associated with delayed attendance among those aged ≥70 years. Delayed attendance was common in these age groups and was in turn associated with increased risk of referral to a GP, indicative of missed opportunities to detect potentially serious eye conditions.

## Supplementary Information

**Figure S1.** Distribution of eye examination intervals among those aged ≥60 years, Northern Ireland, UK. **Table S1.** Individual and household characteristics and associations between characteristics and delayed attendance at routine eye examinations, 2009–2014, among those aged 60 years and over in Northern Ireland, UK. **Table S2.** Individual and household characteristics and associations between characteristics and early attendance at routine eye examinations, 2009–2014, among those aged 60 years and over in Northern Ireland, UK.

## References

[1] Lim JH, Wickremasinghe SS, Xie J *et al*. Delay to treatment and visual outcomes in patients treated with anti-vascular endothelial growth factor for age-related macular degeneration. *Am J Ophthalmol* 2012; 153: 678–686. 22245460 10.1016/j.ajo.2011.09.013 PMC4869322

[2] Rasmussen A, Brandi S, Fuchs J *et al*. Visual outcomes in relation to time to treatment in neovascular age-related macular degeneration. *Acta Ophthalmol* 2015; 93: 616–620. 26073051 10.1111/aos.12781

[3] Echouffo-Tcheugui JB, Ali MK, Roglic G *et al*. Screening intervals for diabetic retinopathy and incidence of visual loss: a systematic review. *Diabet Med* 2013; 30: 1272–1292. 23819487 10.1111/dme.12274

[4] Fung M, Myers P, Wasala P *et al*. A review of 1000 referrals to Walsall's hospital eye service. *J Public Health* 2016; 38: 599–606. 10.1093/pubmed/fdv081 26076700

[5] Davey CJ, Green C & Elliott DB. Assessment of referrals to the hospital eye service by optometrists and GPs in Bradford and Airedale. *Ophthalmic Physiol Opt* 2011; 31: 23–28. 21070302 10.1111/j.1475-1313.2010.00797.x

[6] Pierscionek TJ, Moore JE & Pierscionek BK. Referrals to ophthalmology: optometric and general practice comparison. *Ophthalmic Physiol Opt* 2009; 29: 32–40. 19154278 10.1111/j.1475-1313.2008.00614.x

[7] Department of Health, Social Services and Public Safety. *2014 Northern Ireland Sight Test & Ophthalmic Public Health Survey*. Belfast, UK, 2014. http://www.hscbusiness.hscni.net/pdf/Sight_Test_and_Ophthalmic_Public_Health_Survey_-_Report.pdf (Accessed 11/10/16).

[8] Department of Health Social Services and Public Safety. *2017 Northern Ireland Sight Test and Ophthalmic Public Health Survey*. Belfast, UK, 2017. https://www.health-ni.gov.uk/publications/2017-northern-ireland-sight-test-and-ophthalmic-public-health-survey (Accessed 21/12/18).

[9] Bowling B, Chen SDM & Salmon JF. Outcomes of referrals by community optometrists to a hospital glaucoma service. *Br J Ophthalmol* 2005; 89: 1102–1104. 16113358 10.1136/bjo.2004.064378 PMC1772809

[10] Business Services Organisation. *MOS/275 - Memorandum of Understanding: GOS Sight Tests Intervals*. Business Services Organisation: Belfast, UK, 2012.

[11] Conway L & McLaughlan B. *Older People and Eye Tests*. RNIB, 2007. https://www.rnib.org.uk/sites/default/files/Older%20people%20and%20eye%20tests%20Campaign%20report.pdf (Accessed 21/12/18).

[12] Shickle D & Griffin M. Why don't older adults in England go to have their eyes examined? *Ophthalmic Physiol Opt* 2014; 34: 38–45. 24325433 10.1111/opo.12100

[13] Chou C-F, Sherrod CE, Zhang X *et al*. Barriers to eye care among people aged 40 years and older with diagnosed diabetes, 2006–2010. *Diabetes Care* 2014; 37: 180–188. 24009300 10.2337/dc13-1507 PMC4930070

[14] Biddyr S & Jones A. Preventing sight loss in older people. A qualitative study exploring barriers to the uptake of regular sight tests of older people living in socially deprived communities in South Wales. *Public Health* 2015; 129: 110–116. 25687709 10.1016/j.puhe.2014.10.013

[15] Knight A & Lindfield R. The relationship between socio-economic status and access to eye health services in the UK: a systematic review. *Public Health* 2015; 129: 94–102. 25682906 10.1016/j.puhe.2014.10.011

[16] Shickle D & Farragher TM. Geographical inequalities in uptake of NHS-funded eye examinations: small area analysis of Leeds, UK. *J Public Health* 2015; 37: 337–345. 10.1093/pubmed/fdu039 25015580

[17] Wright DM, O'Reilly D, Azuara-Blanco A *et al*. Impact of car transport availability and drive time on eye examination uptake among adults aged ≥60 years: a record linkage study. *Br J Ophthalmol* 2019; 103: 730–736. 29970390 10.1136/bjophthalmol-2018-312201 PMC6582726

[18] Health and Social Care Board. Developing eyecare partnerships 2012–2017. In: *Improving the Commissioning and Provision of Eyecare Services in Northern Ireland*. Belfast, UK, 2017. http://www.hscboard.hscni.net/our-work/integrated-care/ophthalmic-services/developing-eye-care-partnerships/ (Accessed 09/07/19).

[19] Northern Ireland Statistics and Research Agency. *Northern Ireland Census 2011 Quality Assurance Report*, Belfast, UK, 2015. http://www.nisra.gov.uk/archive/census/2011/evaluation/quality-assurance-report.pdf (Accessed 30/09/15).

[20] Business Services Organisation. *Background Quality Report: Family Practitioner Services Statistics for Northern Ireland - Annual report and quarterly updates*. Belfast, UK, 2018. http://www.hscbusiness.hscni.net/pdf/Background%20Quality%20Report%20-%20FPS%20Statistical%20Compendium%20Final.pdf (Accessed 27/02/20).

[21] NISRA. *Northern Ireland Multiple Deprivation Measure 2010*. 2010. http://www.nisra.gov.uk/deprivation/archive/Updateof2005Measures/NIMDM_2010_Report.pdf (Accessed 27/10/15).

[22] R Core Team. *A Language and Environment for Statistical Computing*. R Foundation for Statistical Computing: Vienna, Austria, 2018. Available from https://www.R-project.org/.

[23] Shah R, Evans BJW & Edgar D. A survey of the availability of state-funded primary eye care in the UK for the very young and very old. *Ophthalmic Physiol Opt* 2007; 27: 473–481. 17718886 10.1111/j.1475-1313.2007.00506.x

[24] Bourne RRA, Flaxman SR, Braithwaite T *et al*. Magnitude, temporal trends, and projections of the global prevalence of blindness and distance and near vision impairment: a systematic review and meta-analysis. *Lancet Glob Health* 2017; 5: e888–e897. 28779882 10.1016/S2214-109X(17)30293-0

[25] Doyal L & Das-Bhaumik RG. Sex, gender and blindness: a new framework for equity. *BMJ Open Ophthalmol* 2018; 3: e000135. 10.1136/bmjophth-2017-000135 PMC6146307 30246151

[26] Dickey H, Ikenwilo D, Norwood P *et al*. Utilisation of eye-care services: the effect of Scotland's free eye examination policy. *Health Policy Amst Neth* 2012; 108: 286–293. 10.1016/j.healthpol.2012.09.006 23063565

[27] Maier PC, Funk J, Schwarzer G *et al*. Treatment of ocular hypertension and open angle glaucoma: meta-analysis of randomised controlled trials. *BMJ* 2005; 331: 134. 15994659 10.1136/bmj.38506.594977.E0 PMC558697

[28] Takahashi H, Ohkubo Y, Sato A *et al*. Relationship between visual prognosis and delay of intravitreal injection of ranibizumab when treating age-related macular degeneration. *Retina* 2015; 35: 1331–1338. 25719984 10.1097/IAE.0000000000000513

