# Supplementary material for: Delayed attendance at routine eye examinations is associated with increased probability of general practitioner referral: a record linkage study in Northern Ireland
Source: Ophthalmic Physiol Opt. 2020 Apr 16;40(3):365–75. doi: 10.1111/opo.12685 (PMC12852151; doi:10.1111/opo.12685)

# Delayed attendance at routine eye examinations is associated with increased probability of general practitioner referral: a record linkage study

David M. Wright, Dermot O’Reilly, Augusto Azuara-Blanco, Raymond Curran, Margaret McMullan, Ruth E. Hogg

# Supplementary material

Table S1. Individual and household characteristics and associations between characteristics and delayed attendance at routine eye examinations, 2009-2014, among those aged 60 years and over in Northern Ireland, UK. Multivariate estimates are adjusted for all variables in the table and main text Table 2.

|  |  |  | **Aged 60-69** |  |  | **Aged ≥ 70** |  |
| --- | --- | --- | --- | --- | --- | --- | --- |
| **Variable** | **Level** | **Total (N = 94,986) (%)** | **Delayed (%)** | **Adjusted RRR (95% CI)^a^** | **Total (N=217,013) (%)** | **Delayed (%)** | **Adjusted RRR (95% CI) ^a^** |
| Highest qualification | No qualifications | 50.9 | 17.8 | 0.94 (0.86, 1.01) | 62.3 | 51.2 | 0.96 (0.93, 0.99) |
|  | Foundation | 6.4 | 20.3 | 1.02 (0.90, 1.15) | 3.8 | 53.6 | 1.08 (1.02, 1.15) |
|  | Vocational | 5.4 | 20.7 | 1.09 (0.95, 1.24) | 4.9 | 52.2 | 1.03 (0.98, 1.09) |
|  | Apprenticeship | 5.0 | 20.8 | 1.00 (0.87, 1.14) | 4.8 | 51.4 | 1.00 (0.95, 1.06) |
|  | Five plus GCSEs | 8.7 | 20.1 | 1.03 (0.92, 1.15) | 5.9 | 53.8 | 1.06 (1.01, 1.12) |
|  | Two plus Alevels | 4.5 | 19.8 | 1.02 (0.89, 1.18) | 3.0 | 53.0 | 1.05 (0.98, 1.12) |
|  | Degree | 19.0 | 18.9 | 1.00 | 15.2 | 51.6 | 1.00 |
| Carer | Provides no unpaid care | 82.4 | 18.6 | 1.00 | 86.6 | 51.5 | 1.00 |
|  | Provides 1 to 19 hours unpaid care a week | 8.3 | 20.3 | 0.96 (0.87, 1.06) | 5.0 | 52.3 | 1.02 (0.97, 1.07) |
|  | Provides 20 to 49 hours unpaid care a week | 2.7 | 21.7 | 1.08 (0.91, 1.27) | 1.9 | 54.7 | 1.12 (1.04, 1.21) |
|  | Provides 50 or more hours unpaid care a week | 6.6 | 18.1 | 1.01 (0.90, 1.13) | 6.4 | 52.1 | 1.03 (0.98, 1.07) |
| Adaptations | Is not adapted for visual difficulties | 99.7 | 18.8 | 1.00 | 99.5 | 51.7 | 1.00 |
|  | Is adapted for visual difficulties | 0.3 | 14.1 | 0.99 (0.57, 1.72) | 0.5 | 44.5 | 1.03 (0.87, 1.21) |
| Income deprivation | 1 (least deprived) | 22.3 | 19.3 | 1.00 | 22.1 | 52.4 | 1.00 |
|  | 2 | 22.3 | 19.3 | 1.01 (0.93, 1.10) | 22.2 | 51.7 | 0.97 (0.94, 1.00) |
|  | 3 | 16.9 | 18.6 | 0.98 (0.90, 1.08) | 17.4 | 51.4 | 0.95 (0.92, 0.99) |
|  | 4 | 19.3 | 18.5 | 1.03 (0.94, 1.13) | 20.0 | 50.9 | 0.96 (0.93, 1.00) |
|  | 5 (most deprived) | 19.3 | 17.8 | 0.96 (0.87, 1.06) | 18.3 | 51.6 | 0.95 (0.92, 0.99) |
| Highest qualification | No qualifications | 50.9 | 17.8 | 0.94 (0.86, 1.01) | 62.3 | 51.2 | 0.96 (0.93, 0.99) |
|  | Foundation | 6.4 | 20.3 | 1.02 (0.90, 1.15) | 3.8 | 53.6 | 1.08 (1.02, 1.15) |
|  | Vocational | 5.4 | 20.7 | 1.09 (0.95, 1.24) | 4.9 | 52.2 | 1.03 (0.98, 1.09) |
|  | Apprenticeship | 5.0 | 20.8 | 1.00 (0.87, 1.14) | 4.8 | 51.4 | 1.00 (0.95, 1.06) |
|  | Five plus GCSEs | 8.7 | 20.1 | 1.03 (0.92, 1.15) | 5.9 | 53.8 | 1.06 (1.01, 1.12) |
|  | Two plus Alevels | 4.5 | 19.8 | 1.02 (0.89, 1.18) | 3.0 | 53.0 | 1.05 (0.98, 1.12) |
|  | Degree | 19.0 | 18.9 | 1.00 | 15.2 | 51.6 | 1.00 |

Abbreviations: RRR, Relative Risk Ratio; CI, Confidence Interval. **^a^** Highlighting indicates cells with 95% Confidence Intervals not overlapping the reference value (blue = below reference; orange = above reference).

Table S2. Individual and household characteristics and associations between characteristics and early attendance at routine eye examinations, 2009-2014, among those aged 60 years and over in Northern Ireland, UK. Multivariate estimates are adjusted for all variables in the table and main text Table 3.

|  |  |  | **Aged 60-69** |  |  | **Aged ≥ 70** |  |
| --- | --- | --- | --- | --- | --- | --- | --- |
| **Variable** | **Level** | **Total (N = 94,986) (%)** | **Early (%)** | **Adjusted RRR (95% CI)^a^** | **Total (N=217,013) (%)** | **Early (%)** | **Adjusted RRR (95% CI) ^a^** |
| Highest qualification | No qualifications | 50.9 | 74.3 | 0.86 (0.80, 0.92) | 62.3 | 23.0 | 0.87 (0.84, 0.90) |
|  | Foundation | 6.4 | 72.1 | 0.87 (0.78, 0.97) | 3.8 | 23.2 | 0.98 (0.92, 1.06) |
|  | Vocational | 5.4 | 72.0 | 0.91 (0.81, 1.03) | 4.9 | 23.7 | 0.97 (0.91, 1.03) |
|  | Apprenticeship | 5.0 | 70.6 | 0.80 (0.71, 0.91) | 4.8 | 21.8 | 0.89 (0.83, 0.95) |
|  | Five plus GCSEs | 8.7 | 72.5 | 0.90 (0.82, 1.00) | 5.9 | 22.8 | 0.96 (0.90, 1.01) |
|  | Two plus Alevels | 4.5 | 72.8 | 0.91 (0.80, 1.04) | 3.0 | 22.6 | 0.95 (0.88, 1.03) |
|  | Degree | 19.0 | 74.0 | 1.00 | 15.2 | 24.0 | 1.00 |
| Carer | Provides no unpaid care | 82.4 | 73.8 | 1.00 | 86.6 | 23.1 | 1.00 |
|  | Provides 1 to 19 hours unpaid care a week | 8.3 | 71.6 | 0.91 (0.84, 1.00) | 5.0 | 23.2 | 1.05 (0.99, 1.11) |
|  | Provides 20 to 49 hours unpaid care a week | 2.7 | 70.1 | 0.90 (0.78, 1.04) | 1.9 | 22.1 | 1.06 (0.97, 1.16) |
|  | Provides 50 or more hours unpaid care a week | 6.6 | 74.5 | 1.03 (0.93, 1.14) | 6.4 | 23.7 | 1.09 (1.03, 1.15) |
| Adaptations | Is not adapted for visual difficulties | 99.7 | 73.5 | 1.00 | 99.5 | 23.1 | 1.00 |
|  | Is adapted for visual difficulties | 0.3 | 79.0 | 1.05 (0.65, 1.69) | 0.5 | 33.0 | 1.38 (1.16, 1.64) |
| Income deprivation | 1 (least deprived) | 22.3 | 73.2 | 1.00 | 22.1 | 23.2 | 1.00 |
|  | 2 | 22.3 | 73.4 | 0.99 (0.92, 1.07) | 22.2 | 23.7 | 1.00 (0.96, 1.04) |
|  | 3 | 16.9 | 73.8 | 0.98 (0.90, 1.06) | 17.4 | 23.4 | 0.95 (0.91, 0.99) |
|  | 4 | 19.3 | 73.9 | 0.98 (0.90, 1.07) | 20.0 | 23.6 | 0.94 (0.90, 0.98) |
|  | 5 (most deprived) | 19.3 | 73.5 | 0.86 (0.79, 0.95) | 18.3 | 21.6 | 0.81 (0.77, 0.85) |

Abbreviations: RRR, Relative Risk Ratio; CI, Confidence Interval. **^a^** Highlighting indicates cells with 95% Confidence Intervals not overlapping the reference value (blue = below reference; orange = above reference).

Figure S1. Distribution of eye examination intervals among those aged ≥60 years, Northern Ireland, UK. The longest interval group contains all intervals >40 months.


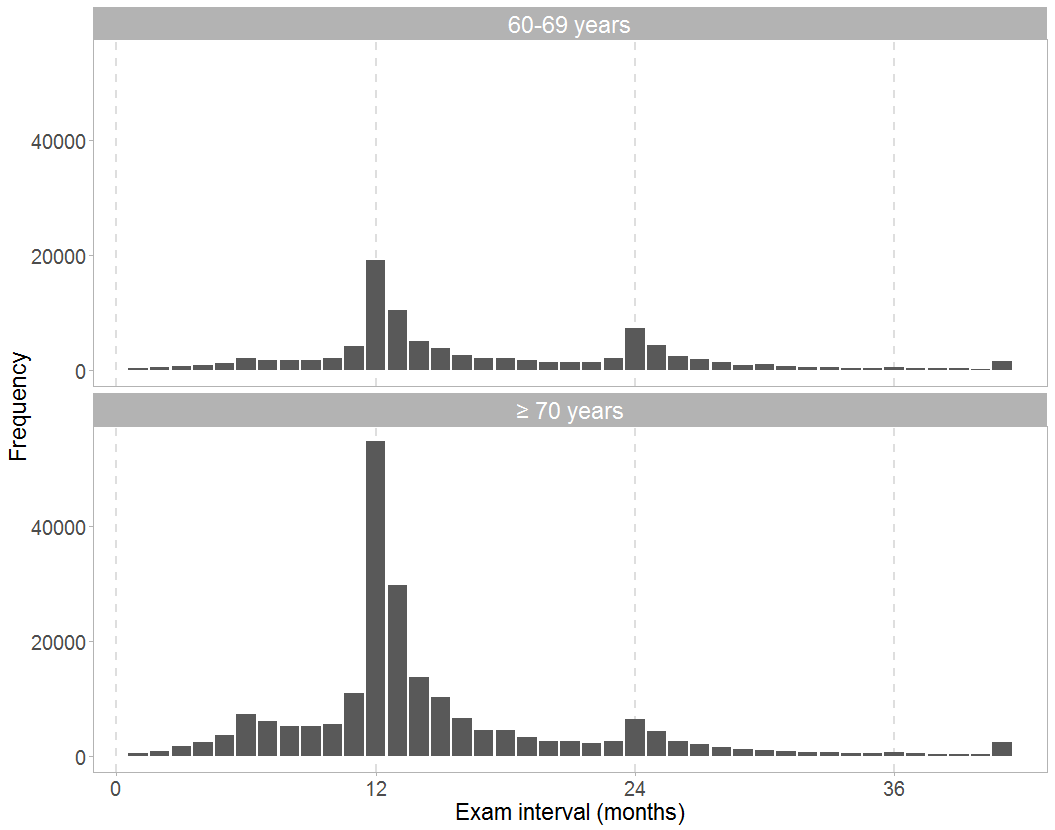

Supplement: Supplementary file 1 — Figure S1. Distribution of eye examination intervals among those aged ≥60 years, Northern Ireland, UK. Table S1. Individual and household characteristics and associations between characteristics and delayed attendance at routine eye examinations, 2009–2014, among those aged 60 years and over in Northern Ireland, UK. Table S2. Individual and household characteristics and associations between characteristics and early attendance at routine eye examinations, 2009–2014, among those aged 60 years and over in Northern Ireland, UK. [file 44402_2020_4003013_MOESM1_ESM.docx]
